# Precise management system for chronic intractable pain patients implanted with spinal cord stimulation based on a remote programming platform: study protocol for a randomized controlled trial (PreMaSy study)

**DOI:** 10.1186/s13063-023-07595-4

**Published:** 2023-09-11

**Authors:** Yuanchen Cheng, Duo Xie, Yan Han, Siying Guo, Zhenxing Sun, Linkai Jing, Weitao Man, Dongkang Liu, Kaiyuan Yang, Dan Lei, Zhe Meng, Huifang Zhang, Guoqin Wang, Weiwei Wu, Guihuai Wang, Yang Lu

**Affiliations:** 1 School of Medicine, Tsinghua University, Beijing, China; 2 Air Force Medical Center PLA, Beijing, China; 3 Department of Neurology Xuanwu Hospital, Capital Medical University, Beijing, China; 4 Department of Neurosurgery, Beijing Tsinghua Changgung Hospital, School of Clinical Medicine, Tsinghua University, Beijing, China; 5 Institute for Precision Medicine, Tsinghua University, Beijing, China

**Keywords:** Chronic intractable pain, Spinal cord stimulation, Remote programming, Mobile health, Randomized controlled trial, Precision medicine

## Abstract

**Background:**

Spinal cord stimulation (SCS) is a surgical technique used in patients with chronic intractable pain, and its effectiveness and safety have been validated by multiple studies. However, to maintain an optimal and steady long-term effect is still challenging. Here, we report a new management paradigm integrating smartphone application and remote programming. Chronic pain patients with SCS implants can monitor their pain status on the phone and change stimulation parameters accordingly. The PreMaSy study is a randomized controlled trial to evaluate the clinical effectiveness and safety of this precise management system.

**Methods:**

Patients with chronic intractable pain will be screened for eligibility, and 82 participants are anticipated to be enrolled in this trial. After the electrode implantation, the stimulation effectiveness will be tested. Participants with a reduction of more than 50% in the visual analog scale (VAS) will receive implantation of an implantable pulse generator and randomized (1:1) into the experimental group or control group. All participants will be asked to take online follow-ups and complete assessments using a smartphone application. Daily pain characteristic assessments and monthly quality of life questionnaires are integrated into the App, and participants will be required to complete these assessments. The daily VAS for pain intensity will be monitored and a threshold will be set based on baseline VAS score. The interventional appointment will be scheduled once the threshold is reached. The primary outcome is the health condition and quality of life assessed by the five-level EuroQol five-dimensional questionnaire (EQ-5D-5L). Utility values of EQ-5D-5L will be assessed at baseline and 1, 3, and 6 months post-operative.

**Discussion:**

The PreMaSy study aims to evaluate the effectiveness and safety of a novel App-based, patient-centered, self-assessment management system for chronic intractable pain. A randomized controlled trial is designed to test the non-inferiority of this precise management system compared to the monthly online follow-ups. It is also expected to yield valuable experiences regarding precision medicine.

**Trial registration:**

ClinicalTrials.gov NCT05761392. Registered on March 07, 2023.

**Supplementary Information:**

The online version contains supplementary material available at 10.1186/s13063-023-07595-4.

## Introduction

As a growing public health concern, chronic pain has an impact on many aspects of patients’ lives. Not only does it affect physical functions and limit daily activities, but it also has a detrimental impact on work, social, and family-related consequences and decreases one’s quality of life [12]. Furthermore, it creates great medical and economic burdens. According to previous reports, the prevalence of chronic pain was approximately 20% in US adults [7] and over 30% in China [53]. The resulting economic burden is therefore enormous. It is estimated that the total cost of pain in the USA in 2008 was between 560 and 635 million dollars [15].

Spinal cord stimulation (SCS) is a technique that uses implanted electrodes to directly stimulate the dorsal columns of the spinal cord. It has been approved and widely used in a variety of pain control applications, for example, failed back surgery syndrome (FBSS) [42], complex regional pain syndrome [50], radiculopathy [51], and diabetic neuropathy [36, 37]. Favorable results were demonstrated by multiple clinical trials [20, 29], confirming the effectiveness of the therapy. However, there are certain hurdles that substantially hampered the real-world applications. First, it is recommended that stimulation paradigms be individualized and modified based on real-time patient feedback in order to maximize the efficacy [38]. Considering the electrode contacts and stimulation parameters such as current, voltage, and frequency, the number of all conceivable combinations is enormous. Second, significant fluctuations are reported in long-term implantations [41]. Thus, a long-term and extensive follow-up plan is often required to maintain a stable stimulation effect. Last but not the least, the disparity of medical service assessment should be taken into account. In China, for instance, only a few cities have qualified SCS-related clinics [23], and the COVID-19 pandemic has largely restricted travel between distant places, which has aggravated this disparity.

Attempts are being made to maintain maximum and stable efficacy with minimal financial and time expenditures by utilizing telemedicine and remote programming. Our group has reported for the first time the clinical application of remote programming for pain patients with a SCS implant using a video-based real-time remote programming system [32]. Our remote sessions allowed us to deliver remote programming operations accurately and safely, while reducing costs and providing convenience for patients.

But we wanted to take one step further. Pain characteristics differ not only across individuals but also within a single individual. For example, the pain intensity may change significantly throughout the day due to weather, mood, or sleep quality, or it may simply vary over time. But these shorter-term shifts are often ignored in both traditional and online programming sessions. Patients have to wait until scheduled follow-up sessions for major adjustments in stimulation parameters. A more precise application-based, patient-centered, self-assessment management scheme has been exploring in a broad range of diseases, for example, Parkinson’s disease [1], atrial fibrillation [17], and asthma [5].

Here, we introduce for the first time a precise management system for pain patients with a SCS implant, which combines self-assessment based on a customized smartphone application (App) and remote programming based on a remote and wireless programming system. In the proposed study, patients who use the precise management system will be compared to those who take conventional online follow-ups. We intend to investigate the effectiveness of this novel system and its application in a broader population.

## Methods

### Trial design

The PreMaSy study is a prospective, double-arm, enrichment-enrollment, randomized-controlled trial (Clinical trial registration ID: NCT05761392) designated to evaluate the effectiveness of the precise management system for chronic intractable pain. The precise management system is a novel App-based, patient-centered, self-assessment modality designed for patients who have chronic intractable pain and are treated with remote and wireless SCS. Compared with a classic RCT, an enrichment-enrollment trial increases the proportion of likely responders in order to maximize the differences between treatment and placebo effects, for example, in many analgetic drug trials [14, 39]. To enroll more “true” responders, the enrichment-enrollment trial shifts the randomization point, and in our instance, only after participants achieve at least a 50% reduction in pain intensity and tolerate SCS well during the test period.

**Fig. 1:**
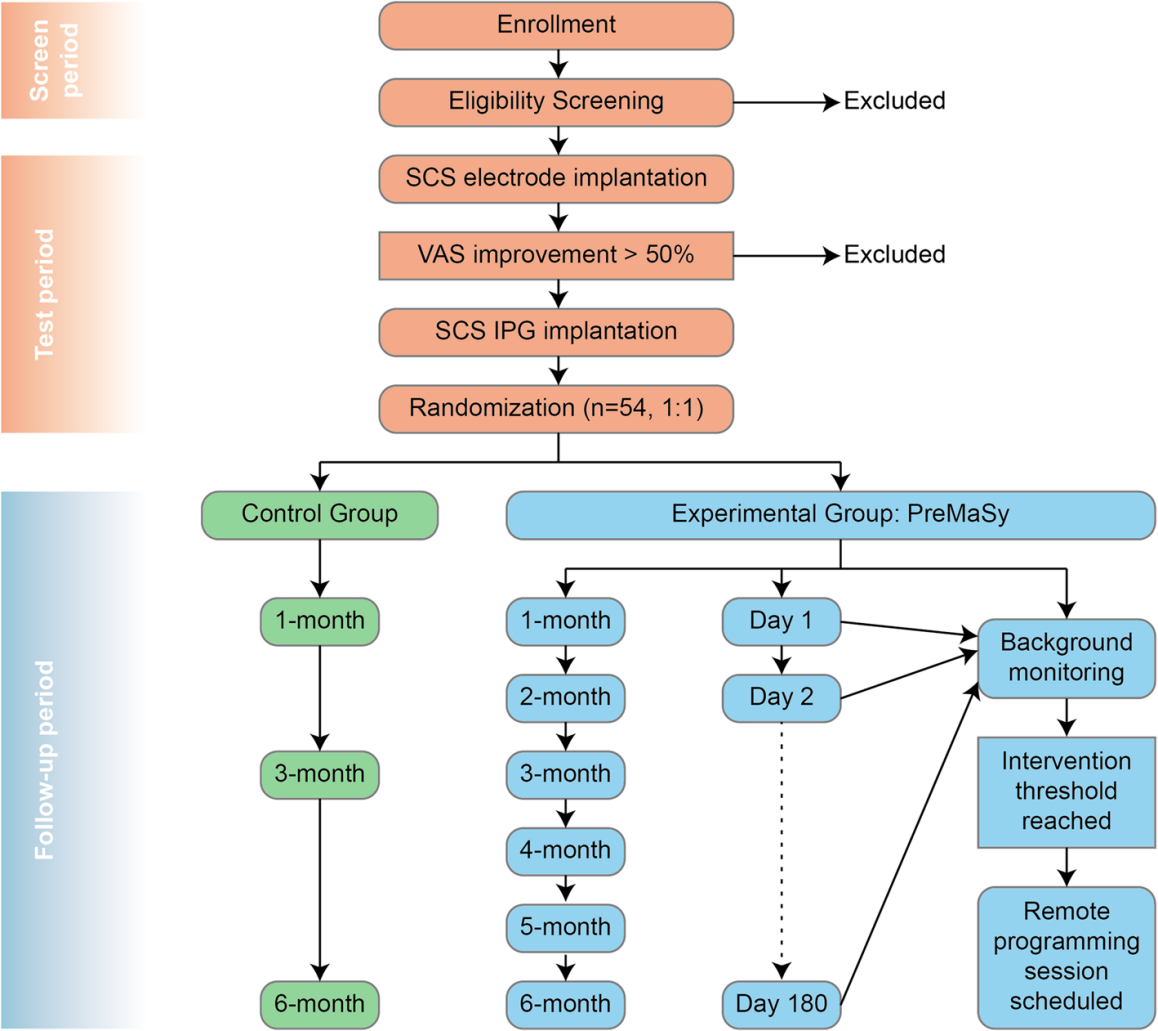
Flow chart of the PreMaSy study

**Fig. 2:**
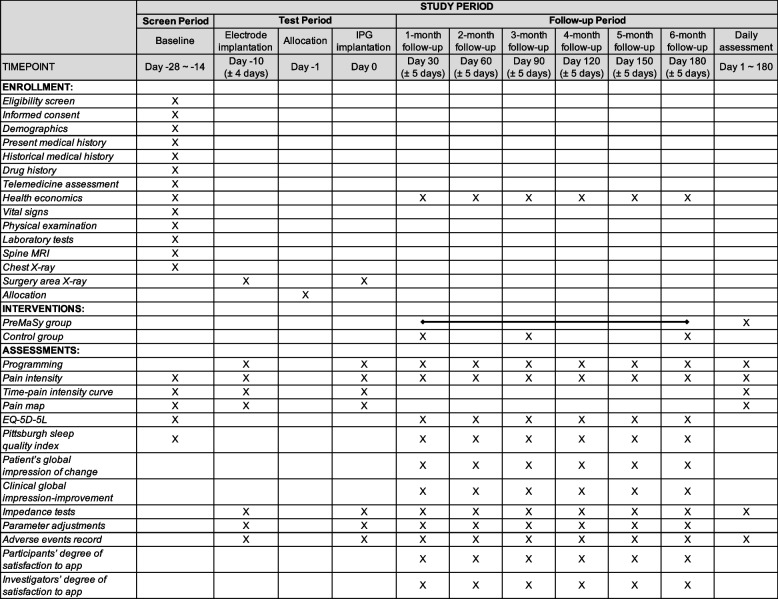
Standard Protocol Items: Recommendations for Interventional Trials (SPIRIT) figure

The timeline of this study is divided into three distinct periods: (1) screen period, (2) test period, and (3) follow-up period (Fig. 1). Patients experiencing chronic intractable pain will be invited to participate in our study. After signing the informed consent, participants will be screened strictly for eligibility based on the inclusion and exclusion criteria (Tables 1 and 2). During the 2-week screen period, admission arrangements will be made and thorough pre-operative examinations will be administered. In addition to collecting basic information including demographics and present and historical medical history, baseline assessments including pain characteristics and quality of life will be documented as well (Fig. 2).

**Table 1:** Inclusion criteria of the PreMaSy study

Inclusion criteria
1. Have been clinically diagnosed with chronic intractable pain for at least 3 months
2. Be unresponsive to or unsatisfied with conservative treatments, including but not limited to oral medication, nerve block, epidural corticosteroids, physical- and psychological rehabilitation therapy, and chiropractic care
3. At enrollment, aged 18 years or older
4. At enrollment, average pain intensity of at least 50 out of 100 mm on a visual analog scale
5. Be voluntary to take the trial and sign the informed consent
6. Have good compliance and ability to complete post-operative follow-ups
7. Have the basic ability to read and use a mobile phone or have a caregiver who can

**Table 2:** Exclusion criteria of the PreMaSy study

Exclusion criteria
1. Have bleeding complications or coagulation disorders
2. Have mental or cognitive disorders leading to inability to complete implantation surgery or post-operative follow-up
3. Have issues with the spinal cord or vertebrates that are not suitable for implantation surgery
4. Have systemic active infections or local infections at the anticipated surgery area
5. Be pregnant, breast-feeding, plan to be pregnant, or unwilling to use contraceptive methods
6. Have metastatic tumors or untreated malignancies
7. Have a life expectancy of less than 1 year
8. Have already provided with a medication pump and/or other implanted devices
9. Require the use of MRI and/or thermo-therapy
10. Be heavily addicted to alcohol and/or drugs
11. Have an improvement in VAS of less than 50% or unable to tolerance SCS during the test period
12. Unable to complete long-term online follow-ups because of hardware issues such as internet, mobile phones, and so on

Participants will then undertake the SCS electrode implantation and enter the 10-day (± 4) test period. Pain intensity will be monitored during this time. Only participants who have an improvement of no less than 50% in visual analog scale (VAS) will undertake the implantable pulse generator (IPG) implantation. After the IPG implantation, participants will be randomized (1:1) into one of the two groups: the experimental group (EG) with PreMaSy or the control group (CG) with conventional follow-ups. The randomization will be generated by computers and concealed from the recruitment team and data analysis team. The participants and their physicians, however, will not be blinded from their own randomization results, because the consequent assessments are dependent on which group they are in. The detailed assessments of the two groups are shown in Fig. 2. Participants in CG will take follow-ups only at 1, 3, and 6 months post-operative, while participants in EG will take additional monthly quality of life assessments and daily pain characteristics assessments, which can be done in a customized App. These results will be automatically monitored and timely interventional appointment will be scheduled whenever the intervention threshold is reached.

The PreMaSy study protocol was written in accordance with the Standard Protocol Items: Recommendations for Interventional Trials Statement (SPIRIT). A SPIRIT checklist is included in Additional file 1. The trial will be carried out according to the principles of the Declaration of Helsinki (Edinburgh version, 2000).

### Participants

#### Study population

The study will be primarily conducted in the Neurosurgery Center of Beijing Tsinghua Changgung Hospital. Additional centers may be added as the study progresses. The protocol has been approved by the Clinical Trial Ethics Committee of Beijing Tsinghua Changgung Hospital (approval number: 22442–4-02). It is anticipated that the enrollment will end prior to September 2024. And invitations will be sent to our clinic patients experiencing chronic intractable pain during that time.

#### Inclusion criteria

The inclusion criteria are shown in Table 1.

#### Exclusion criteria

The exclusion criteria are shown in Table 2.

### App design

Our research team in cooperation with Beijing Pinchi Software Development Co., Ltd. developed an App that integrates the wireless control of the stimulation device and the follow-up assessments. The App can connect wirelessly to the IPG device based on our previously developed remote and wireless SCS system [32]. Four sets of personalized stimulation parameters will be programmed to optimize the analgesic effect and stored in the App before participants are discharged. Participants will be given complete control over the stimulation, including turning it on and off and changing the parameter set in use according to their preferences.

The assessment module was designed as a crucial element in the precise management system. Four major sections are included: (1) daily assessments, (2) monthly assessments, (3) hardware-related issues, and (4) satisfaction with App usage.

**Fig. 3:**
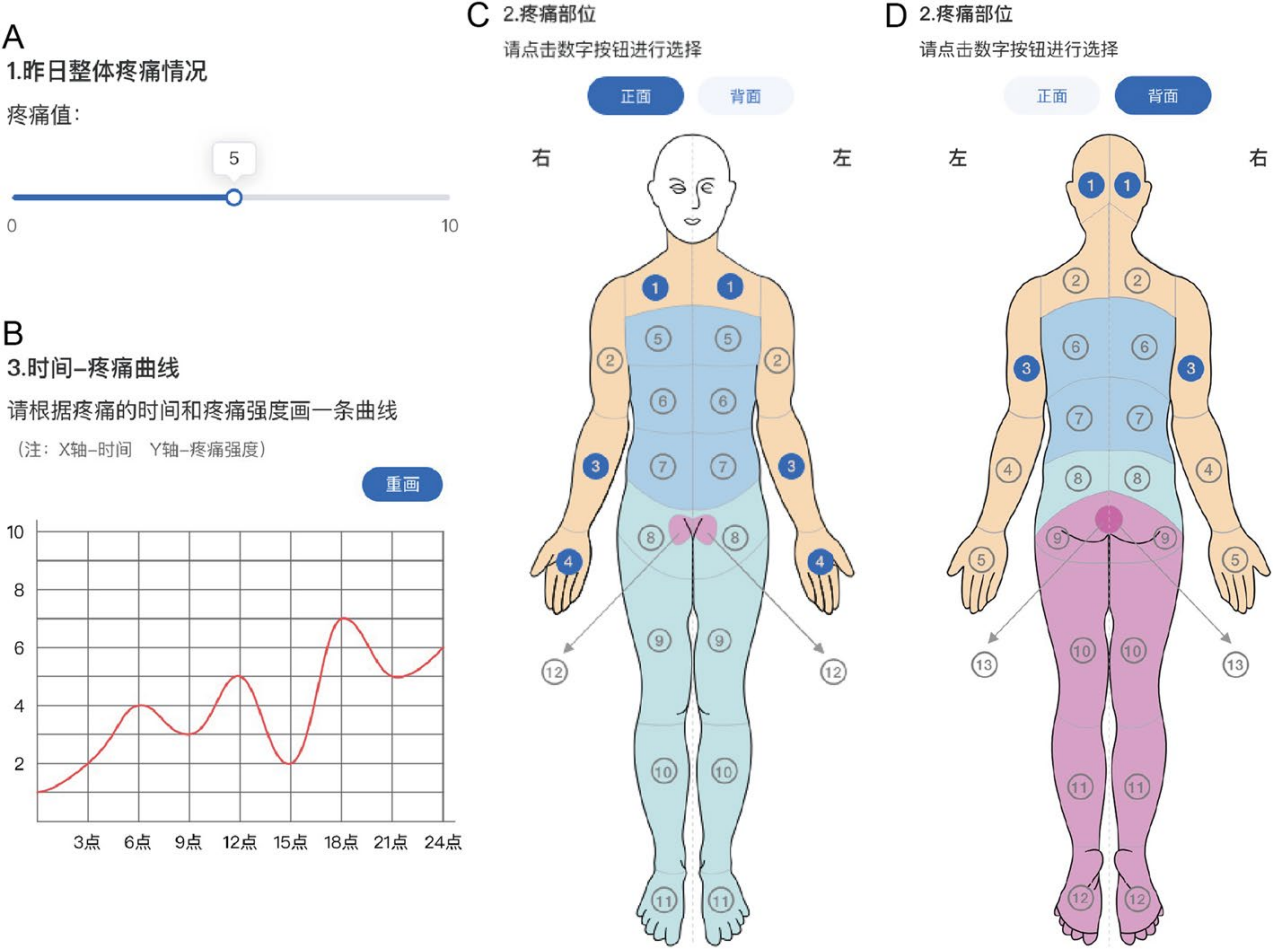
The daily assessments designed for the App. **A** Slider of VAS for general pain intensity. The current position indicates 5.0 out of 10.0. **B** Time-intensity curve for pain temporal characteristics. The *x*-axis depicts 24 h in a day in 3-h increments, and the *y*-axis depicts the pain intensity. The red curve simulates a patient’s hand-drawn curve. **C**, **D** Simplified pain map based on the dermatome pattern. There are a total of 25 pairs of body area presented, including 12 front (**C**) and 13 back (**D**). The indicators highlighted in blue simulate selections from an imaginative candidate

The daily assessment section primarily focuses on the evaluation of multiple pain characteristics, including intensity, temporal, and spatial features. Pain intensity is measured by a VAS slider customized to mobile phone screen size (Fig. 3A) [44]. To measure the temporal features, we designed a hand-drawn time-intensity curve (Fig. 3B). The *x*-axis of the curve represents 24 h in a day, while the *y*-axis represents the VAS score of pain intensity. The participants are asked to recall their pain experience from the previous day and draw a curve to depict the fluctuations in pain intensity over time. As for the spatial features, we referred to the pain area drawing in the Brief Pain Inventory (BPI) and designed a version that is touch-screen compatible. In order to standardize the pain map, the front and rear body projections were separated according to the dermatome pattern. However, the pattern was too detailed to be identified accurately on a smartphone display. Therefore, we consolidated certain consecutive dermatomes and generated 25 gross selection areas (Fig. 3C, D).

The monthly assessment section, on the other hand, focuses on the long-term pain-related quality of life. The Five-Level EuroQol Five-Dimensional Questionnaire (EQ-5D-5L) is a widely used instrument for assessing general health that consists of mobility, self-care, usual activities, pain/discomfort, and anxiety/depression [21]. We used the published value set for China to evaluate various health statuses [34]. The EQ-VAS is used as well for the global health condition. Sleep quality is assessed by the Pittsburgh Sleep Quality Index (PSQI) with 18 items covering seven categories [4]. Patients’ Global Impression of Change (PGIC) and Clinical Global Impressions-Improvements (CGI-I) are two seven-level questionnaires that measure the perceptions of the improvements in the participants’ overall health conditions, respectively from their own [45] and their physicians [3].

Impedance tests, parameter adjustments, adverse events (AEs), and other hardware-related issues are collected. Participants and their physicians are also asked to take satisfaction surveys based on their App usage experiences and encouraged to provide feedback. All aforementioned assessment modules will be activated automatically when the profile of a participant is established, and in order to enhance compliance, notifications of incomplete assessments will be sent at a set time.

### Interventions

During the test period, electrode implantation and IPG implantation will be undertaken in patients who qualify. After surgery, stimulation parameters will be optimized for analgesia. In our prior work, we have described the implantation surgery, stimulation device, and programming procedure in detail [31]. The aforementioned App will be utilized for subsequent follow-ups and pain management. Upon enrollment, an App account will be assigned to each participant. They will be instructed to activate the account and fill up the profile. As soon as they undergo the implantation procedure and are randomly assigned to either EG or CG, the App will only display contents exclusively to their group.

For participants in CG, they will be scheduled for follow-ups at conventional intervals, i.e., 1, 3, and 6 months post-operative. They can only access monthly assessments during the corresponding months. For participants in EG, on the other hand, the precise management system will be implemented. In addition to the standard follow-ups, they will be asked to complete every monthly assessment throughout the 6-month follow-up period. They will have access to the daily assessments in which they will be instructed to record and track their pain characteristics. Pain intensity VAS data collected after the electrode implantation and prior to the IPG implantation will be averaged and served as the baseline. The interventional threshold will be a minimum clinically significant difference (MCSD) of 20 mm (out of 100 mm VAS) [27] added to the baseline value. The system will monitor each participant’s daily VAS score automatically. If the score surpasses the threshold three times in a row, an alarm will be sent to their physicians and a remote programming appointment will be scheduled.

To improve adherence, the background system will monitor the assessments’ completion status, and specialists will be allocated to inquire about the incompletion. Other concomitant care including medications are only permitted by approval of investigators. Both CG and EG participants have the same access to report hardware-related issues and satisfaction with App usage. In the case of adverse events (AEs) or unexpected worsening, they may also schedule an urgent appointment.

### Outcomes

#### Primary outcome

As previously stated, EQ-5D-5L is a questionnaire which measures five dimensions. It depicts different health conditions as five-digit numbers corresponding to unique utility values. The primary outcome of the PreMaSy study is the change of EQ-5D-5L utility values between CG and EG at the 6-month follow-up compared to baseline values. After passing the eligibility screening, they will be required to fill up an EQ-5Q-5L questionnaire based on their pain experience prior to enrollment, and this value will serve as the baseline. The primary outcome formula is:

$$\left({\mathrm{EQ}}_{\mathrm{EG}6}-{\mathrm{EQ}}_{\mathrm{EGbase}}\right)-\left({\mathrm{EQ}}_{\mathrm{CG}6}-{\mathrm{EQ}}_{\mathrm{CGbase}}\right)$$

#### Secondary outcomes and safety outcomes

The differences in EQ-5D-5L utility values at 1- and 3-month follow-ups compared to baseline will be included in the secondary outcomes. Other secondary outcomes include changes in pain intensity VAS, time-intensity curve, pain map, EQ-VAS score, PSQI, PGIC, CGI-I, and satisfaction with App usage. The occurrence rate of AEs and severe AEs (SAEs) will be used as safety outcomes.

### Statistical methods

#### Sample size

The primary comparison in this study is the difference between EG and CG in quality of life score measuring by EQ-5D-5L. The EQ-5D-5L difference is assumed to be 0.53 with a standard deviation (SD) of 0.69 based on previous studies into the effectiveness of App-based therapy for chronic pain [40]. A sample size of 82 (41 per group) is calculated by the following formula:

$$n=\frac{(1+r){({Z}_{\alpha }+{Z}_{1-\beta })}^{2}{\sigma }^{2}}{r{(\left|D\right|+\Delta )}^{2}}$$

$Z$ is the quantile of the standard normal distribution, $\alpha$ is the first-class error level, $\beta$ is the second-class error level for the statistical test, and $r$ is the allocation ratio, which is 1:1 in this equal allocation trial. Here, to detect the significant difference at a significant level of 0.05 with 80% power using a two-tailed* t* test, ${Z}_{\alpha }$ and ${Z}_{1-\beta }$ are determined as 1.96 ($\alpha$ = 0.05) and 0.84 ($\beta$ = 0.2), respectively. $\sigma$ is the expected SD of the outcome, and $\left|D\right|$ is the expected mean difference of the two groups. The non-inferiority limit is set at 10% [10], and $\Delta$ is 0.053. The sample size per group is calculated with the above formula, and the given value is 22. With the assumption of a 60% “true” responding rate and a 10% possible attrition rate, the final calculation of the sample size is 41 pairs ($N$ = 82).

#### Primary and additional analyses

The primary effectiveness analysis is the 6-month non-inferiority of the within-group difference between CG and EG for the overall health-related quality of life measured by EQ-5D-5L. Non-inferiority will be tested between the two groups at a one-sided significance of 0.05, and if the non-inferiority of the primary endpoint was achieved, superiority will be tested at a one-sided significance of 0.025.

For secondary endpoints, non-inferiority will be also tested with a 10% non-inferiority margin and a one-sided significance of 0.05. Secondary endpoints include changes from baseline of EQ-5D-5L scores in 1 and 3 months, VAS scores, time-intensity curves, pain map, EQ-VAS scores, PSQI, PGIC, CGI-I, and satisfaction with App usage. Continuous variables will be summarized using means and standard error, and categorical variables will be summarized using frequency distributions. Parametric tests (e.g., *t* test and ANOVA) will be performed with data of normal distributions. Non-parametric tests (e.g., Wilcoxon rank-sum test and Mann–Whitney test) will be performed if parametric tests are not indicated. All statistical analyses will be performed in a validated statistical software package (e.g., SAS or SPSS).

### Data collection and management

Each participant will be given a unique account number for the App. During hospitalization, all baseline data will be collected, and after the implantation, all participants will be instructed by qualified staff to complete the daily pain assessments. Additional instructions will be provided before the discharge. All data will be uploaded and stored in a cloud-based internet platform, and the transmission will be encrypted to ensure security and confidentiality. All the staff participating in the trial must complete the full training course, which includes participant enrollment, assessment completion on the mobile App, surgery technique, programming demonstration, database demonstration, and CRF completion. Participants and at least one caregiver should be familiar with the mobile App and remote programming. Initial data monitoring will be performed by the investigator himself/herself to ensure authenticity and completeness. Then the certified clinical research associate (CRA) will monitor, audit, and assure compliance with the protocol, GCP, and regulatory requirements during data collection.

## Discussion

SCS is one of the surgical approaches for patients with chronic intractable pain. It is sometimes seen as “the last resort therapy” for those who do not respond well to medications or other non-surgical treatments. Since the first implanted SCS system in 1967 [49], significant development has been established within the past six decades.

Conventional frequency SCS (CF-SCS) delivers a frequency of 10 to 300 Hz. It targets the spinal inhibitory GABA system and usually generates parenthesias [16]. High-frequency SCS (HF-SCS) delivers a frequency of 5 to 10 kHz, and the mechanism is similar to CF-SCS but more robust. Multiple studies have verified the efficacy and safety of HF-SCS in relieving chronic back and leg pain (CBLP) [33, 48, 52] and painful diabetic neuropathy (PDN) [46]. Results from a 24-month RCT indicated the superiority of HF-SCS over CF-SCS in long-term therapy [25]. Another emerging waveform is the non-tonic burst stimulation. For burst programming, five pulses are delivered within 1 ms at a frequency of 40 Hz. Burst SCS is also safe and effective, and it has been proven to be superior to tonic CF-SCS in the treatment of chronic pain [8, 22]. There are other new current delivery methods that adjust stimulation dynamically according to the neurophysiological response to SCS, for example, evoked compound action potential (ECAP) SCS [11] and closed-loop [36, 37].

There are still challenges in the SCS field. The first one is to optimize the efficacy, which requires the personalization of stimulation paradigms according to each patient’s need. But the efficacy can be affected by multiple variables, including but not limited to stimulation parameters, lead location, patient anatomy, and pain pattern [20]. Personalization could be quite difficult due to the intricacy of the stimulation paradigm and the heterogeneity of the patients. The second one is to sustain efficacy. Long-term efficacy has been demonstrated in several studies [25]. However, post-operative fluctuations due to electrode migration, electrode malposition, incision site infection, or loss of therapeutic effect were reported [29, 43]. In addition, patients often suffer fluctuations throughout the day [41], which significantly lowers their quality of life [18]. The third one is the disparity in patient access to SCS-related medical services. It is shown that economically developed regions are more likely to have better chronic pain diagnosis and treatment and vice versa [23]. This disparity makes it more difficult and costly for patients who live far from a SCS clinic to receive therapy.

To address these challenges, efforts were made in the developments of neurostimulation technologies [9]. Advances in Bluetooth technology allow wireless communication between leads and IPG, enabling remote programming and device miniaturization. The feasibility of remote programming is validated by a randomized trial that remotely instructed nonexpert individuals to program the neuromodulation device successfully [38]. Telemedicine is also a new and promising technology that provides remote healthcare service to rural or medically underserved regions [35]. It is considered as an alternative to the conventional outpatient clinic since it improves equity of access to healthcare while also increasing the consistency and quality of service [6]. Combined with telemedicine and remote programming, geographical barriers can be broken and patients can take programming sessions in their homes [32]. In the meantime, eTools and smartphone Apps are targeting more aspects including health care, education, and self-monitoring [2]. Since the COVID-19 pandemic, access to traditional face-to-face service has been largely limited, which in turn promotes transformation toward telemedicine to meet the increasing need for remote programming [26, 30].

Efforts on developing such Apps have already been made in the management of chronic pain [13, 28]. However, the use of SCS was not covered in these studies. In our previous studies, we demonstrated the high demands for remote programming among patients with chronic intractable pain in China [19] and established the safety and effectiveness of a remote wireless programming system [32]. By integrating daily and monthly assessments into a customized smartphone App, we aim to establish an App-based, patient-centered, self-assessment precise management system.

In our customized App, information on patient education and assessments of semiological monitoring are provided as basic functions, which is consistent with other Apps [13, 28]. For semiological monitoring, daily and monthly assessments are provided. Daily assessments of pain focus on three different perspectives, namely intensity, temporal, and spatial characteristics. Monthly assessments, on the other hand, focus on the general health-related quality of life. These assessments instruct participants to focus on their pain experiences and how they alter with stimulation. Daily fluctuations will be captured, and interventions will be delivered in time based on this information. In order to maximize the potential of smartphone Apps [47], participants are also provided with a selection of stimulation paradigms, including two CF-SCS sets and two HF-SCS sets. A real-world investigation indicated that patients may have different stimulation paradigms preferences [24].

There are some limitations in this study. The primary objective is to determine whether this new precise management system is noninferior to conventional online follow-ups. However, the evidence of App effectiveness is still absent. The effect due to the different intensities of App usage cannot be fully interpreted. Another limitation is that the intervention cannot be blinded because it makes direct changes in patients’ lifestyle and relies on their feedbacks. In addition, the overall duration of follow-ups is only 6 months, which is insufficient for exploring long-term consequences and reducing placebo effects.

## Trial status

Protocol version 1.3 (March 25, 2023). The recruitment began in September 2022 and is expected to be completed in September 2024.

### Supplementary Information

**Additional file 1. **Reporting checklist for protocol of a clinical trial.

## Data Availability

This is a study protocol only. The final trial dataset will be kept strictly confidential and can only be accessed by members of the trial team.

## Abbreviations

AE(s): Adverse event(s)
App(s): Application(s)
BPI: Brief Pain Inventory
CBLP: Chronic back and leg pain
CF-SCS: Conventional frequency spinal cord stimulation
CG: Control group
CGI-I: Clinical Global Impressions-Improvements
CRA: Clinical research associate
CRF: Case report form
EACP: Evoked compound action potential
EG: Experimental group
EQ-5D-5L: Five-Level EuroQol Five-Dimensional Questionnaire
FBSS: Failed back surgery syndrome
GCP: Good Clinical Practice
HF-SCS: High-frequency spinal cord stimulation
IPG: Implantable pulse generator
MCSD: Minimum clinically significant difference
PDN: Painful diabetic neuropathy
PGIC: Patients’ Global Impression of Change
PSQI: Pittsburgh Sleep Quality Index
RCT: Randomized controlled trial
SAE(s): Severe adverse event(s)
SCS: Spinal cord stimulation
SD: Standard deviation
VAS: Visual analog scale
